# Neuroimaging signatures of facial onset sensory and motor neuronopathy (FOSMN)

**DOI:** 10.1007/s00415-026-14174-5

**Published:** 2026-10-05

**Authors:** Angela Rosenbohm, Francisco Herrera-Angeles, Hans-Jürgen Huppertz, Karl Georg Haeusler, Jochen H. Weishaupt, Hans-Peter Müller, Jan Kassubek

**Affiliations:** 1 Department of Neurology, University Hospital Ulm, Ulm, Germany; 2 Swiss Epilepsy Clinic, Klinik Lengg, Zurich, Switzerland; 3 Department of Diagnostic and Interventional Radiology, University Hospital Ulm, Oberer Eselsberg 45, 89081 Ulm, Germany; 4 Department of Nuclear Medicine, University Hospital Ulm, Ulm, Germany

**Keywords:** Facial onset sensory and motor neuronopathy, Amyotrophic lateral sclerosis, Magnetic resonance imaging, Motor neuron disease, Atlas-based volumetry, Diffusion tensor imaging, Neuroimaging

## Abstract

Facial onset sensory and motor neuronopathy (FOSMN) is an exceptionally rare condition characterized by progressive facial paresthesia and the subsequent appearance of bulbar symptoms with rostro-caudal motor and sensory deficits sometimes extending to the hand muscles. The pathophysiological understanding of this disorder is very limited. In order to identify patterns of neuroanatomical involvement within the brain, we applied advanced magnetic resonance imaging-based techniques to a group of six patients with the diagnosis of FOSMN, using unbiased analysis techniques with atlas-based volumetry of volume-rendering T1-weighted data and whole brain-based spatial statistics of diffusion tensor imaging (DTI). The volumetric data showed significant global atrophy with prominent involvement of the medulla oblongata, while DTI demonstrated significant decreases in the right-hemispheric frontal lobe and bilaterally along the superior and inferior longitudinal fasciculi. In summary, this application of volumetry and DTI to a group of FOSMN patients demonstrated, beyond global brain atrophy as a correlate of a systemic disease process, regional atrophy in the brainstem/medulla oblongata, together with tract alterations of polymodal frontotemporoparietal interconnections. Future neuroimaging studies in larger patient groups of FOSMN are needed for detailed phenotype–neuroimaging correlations.

## Introduction

Facial onset sensory and motor neuronopathy (FOSMN) is an exceptionally rare condition, with only approximately 100 cases documented worldwide since its first description in 2006 [1–3]. FOSMN typically presents initially with unilateral facial paresthesia followed by muscle weakness that gradually progresses over a span of 2 to 6 years, characteristically spreading to encompass the entire face, scalp, and upper limbs [4], with the subsequent appearance of prominent bulbar symptoms, such as orofacial dysphagia, documented up to 6 years post-onset [5, 6]. A highly characteristic feature of the FOSMN syndrome is the atrophy of the temporal and masseter muscles. Nonetheless, several reports have documented cases where bulbar symptoms served as the presenting feature [3, 8–11]. Both motor and sensory deficits typically evolve in a strict rostro-caudal trajectory, eventually extending to the intrinsic hand muscles. While the progression of sensory impairment is well-documented, the evolution of motor deficits is frequently more prominent [9, 12, 13], with pareses and profound muscle atrophy noted as late as 10 years after initial symptom onset [1]. Notably, lower motor neuron signs—predominantly muscle atrophy—are a universal finding across all documented FOSMN cases [2, 12].

Diagnostic workup relies heavily on electrophysiology: a prolonged or absent blink reflex is a key clinical hallmark, while needle EMG shows neurogenic denervation and reinnervation across cranial, cervical, and thoracic levels, depending on the clinical involvement. Sensory nerve conduction studies typically show reduced SNAP amplitudes in upper limbs. Brain MRI, CSF analysis, and lab work are generally unremarkable, though MRI may demonstrate tongue or cord atrophy, and rare ALS-related genetic variants may be present [2, 7, 14].

Although the precise etiology of this disorder remains elusive, growing evidence suggests some clinical and pathophysiological overlap with both amyotrophic lateral sclerosis (ALS) and frontotemporal dementia (FTD): the first due to motor symptoms with bulbar syndrome and pareses/muscle atrophy the latter, as cognitive deficits and behavioral changes became apparent in a subset of patients [2]. This link is underscored by recent genetic studies identifying mutations in ALS-associated genes, including *SOD1*, *TARDBP*, and *SQSTM1* [7, 14]. To date, universally accepted diagnostic criteria have not been established, although diminished or absent corneal reflexes alongside abnormalities in blink reflex testing have been proposed as key diagnostic markers [15].

The main initial differential diagnoses for FOSMN include bulbar-onset amyotrophic lateral sclerosis (ALS), Bickerstaff brainstem encephalitis (BBE), and Miller Fisher syndrome (MFS). While bulbar-onset ALS shares lower motor neuron features such as severe dysphagia, dysarthria, and tongue atrophy, it presents primarily as a pure motor disorder, lacking the prominent facial sensory loss and diminished corneal reflexes characteristic of FOSMN. Conversely, BBE and MFS can mirror cranial nerve involvement but are distinguished by their acute or subacute postinfectious presentation, presence of ophthalmoplegia and ataxia, contrasting sharply with the slow neurodegenerative course of FOSMN [16, 17].

Previously, routine magnetic resonance imaging (MRI) findings in patients with FOSMN are predominantly reported to be unremarkable; however, postmortem neuropathological examinations have revealed significant atrophy of the trigeminal nerve roots alongside degenerative changes within both the sensory and motor nuclei of the trigeminal nerve [18]. In addition, postmortem analyses provide definitive evidence of substantial anterior horn cell loss [19], further reinforcing the systemic motor neuron involvement. Consequently, the objective of the current study was to comprehensively delineate the spectrum of cerebral alterations of this entity in vivo by grey matter (GM) and white matter (WM) analysis by use of advanced MRI techniques.

## Methods

### Subjects and patient characteristics

All incident patients with FOSMN who presented to the Department of Neurology at the University Hospital Ulm, Ulm, Germany between 2016–2026 were included in this monocentric observational study. Six consecutive FOSMN patients (4 males/2 females, mean age 64.2 ± 16.2 years, range 48–84 years) consented to undergo MRI with DTI analysis.

FOSMN patients were compared with a group of 30 age- and sex-matched healthy controls (20 males/10 females, mean age 64.0 ± 4.3 years, range 54–78 years). Gross brain pathology, including vascular brain alterations, was excluded by conventional MRI. All controls lacked a family history of neuromuscular disease and had no history of neurological, psychiatric, or other major medical illnesses. The study has been approved by the Ethics Committee of the University of Ulm (references #19/12 and #20/12) and was conducted in accordance with the ethical standards laid down in the 1964 Declaration of Helsinki and its later amendments. Written informed consent was obtained from all individual participants included in the study.

### MRI acquisition protocol

MRI scanning was performed on a 1.5 Tesla Magnetom Symphony (Siemens Medical, Erlangen, Germany); the DTI study protocol A (echo time (TE)/repetition time (TR), 95 ms/8000 ms) contained 52 volumes, including 4 b=0 (matrix 128 × 128 × 64, resolution 2.0 × 2.0 × 2.8 mm^3^), DTI study protocol B (TE/TR, 102 ms/8700 ms) contained 62 volumes, including 2 b=0 (matrix 128 × 128 × 64, resolution 2.5 × 2.5 × 2.5 mm^3^). In this retrospective study, 2 FOSMN patients were recorded with protocol A, 4 with protocol B; 11 controls were recorded with protocol A, 19 with protocol B.

### Structural MRI analysis: atlas-based volumetry (ABV)

The T1-weighted data were processed using MATLAB (version R2021b, The Mathworks, USA) and the Statistical Parametric Mapping 12 (SPM12) software (Wellcome Trust Center for Neuroimaging, London, UK, www.fil.ion.ucl.ac.uk/spm), following a standardized processing pipeline for atlas-based volumetry (ABV) [20, 21]. ABV has already been successfully applied to several neurodegenerative conditions in previous studies (e.g., Refs. [22, 23]). In short, the processing steps included: (i) segmentation into gray matter, white matter, and cerebrospinal fluid (CSF) compartments, (ii) stereotaxic normalization into MNI space, and (iii) volumetric assessment using voxel-by-voxel multiplication and subsequent integration of normalized modulated component images (GM, WM, or CSF) with predefined masks from standardized brain atlases (here, LONI Probabilistic Brain Atlas [24]). All volumetric results were linearly standardized to the mean intracranial volume (ICV) of controls. Group-level differences were provided as z values.

### DTI data analysis

The software *Tensor Imaging and Fiber Tracking* (TIFT) [25] was used for data analysis; details of the algorithms have been described in detail previously [26]. In short: after stereotaxic normalization to the Montreal Neurological Institute (MNI) space, fractional anisotropy (FA) to analyze white matter microstructure. Prior to correction for the covariate age, a Gaussian filter of 8 mm full width at half maximum was applied for smoothing of FA maps. Smoothed FA maps were harmonized between protocols A and B. Voxel-wise statistical comparison of the DTI metrics was performed by Welch test to detect alterations between the subject groups (whole brain-based spatial statistics, WBSS); voxels with FA values below 0.2 were not considered for statistical comparison, since cortical grey matter shows FA values up to 0.2 [27]. Correction for multiple comparisons was performed using the false-discovery-rate (FDR) algorithm at *p* < 0.05 [28]; further reduction of the alpha error was performed by a spatial correlation algorithm eliminating small isolated groups of voxels in the size range of the smoothing kernel (8 mm) leading to a threshold cluster size of 512 voxels.

## Results

### Clinical characterization

We examined six patients with clinically confirmed FOSMN (4 m/2f, mean age at presentation 64.2 ± 16.2, range 48–84 years) (Table 1). The initial clinical manifestation was facial numbness in three patients, oropharyngeal dysphagia in one patient, paresis of the masticatory muscles in one patient, and weakness of the right upper limb due to paresis of the forearm flexor muscles in one patient. Oropharyngeal dysphagia was confirmed by Fiberoptic Endoscopic Evaluation of Swallowing (FEES) in five patients. Two patients required percutaneous endoscopic gastrostomy (PEG) placement. Two patients developed upper-limb paresis, with a mean interval from diagnosis to the onset of paresis of 48 months (range, 12 to 84 months). The mean follow-up duration in five patients was 4.1 years (range, 5 months to 10 years). P06 who died after implantation of PEG had a well-documented 12-month time course before admission to our hospital and fulfilled all clinical criteria of the disease with respect to the longitudinal course and the clinical status at admission.

**Table 1:** FOSMN patients’ characteristics

Patient number	P01	P02	P03	P04	P05	P06
Sex	m	f	m	m	m	f
Age at onset/years	46	51	74	40	54	84
Disease duration at MRI/months	50	3	25	>200	21	12
Initial manifestation	Facial numbness	Facial numbness	Masticatory muscle paresis	Right upper-limb weakness (forearm flexors)	Facial numbness	Oropharyngeal dysphagia
Sensory deficits face	Yes	Yes	Yes	No	Yes	Yes
Dysphagia diagnosis/PEG placed or indicated	FEES/yes	FEES/no	FEES/no	Clinical/no	FEES/no	FEES/yes
Upper-limb paresis	Yes	Yes	No	Yes	No	No
lLower-limb paresis	No	No	No	No	No	No
Upper motor neuron affection	No	No	No	No	Yes	No
Follow-up	10 years	5 months	3 years	6 years	1 year	0 months
Blink reflex abnormal	–	–	–	–	–	Yes
EMG: denervation	No	Yes	Yes	Yes	Yes	Yes
NCS: SNAP amplitude reduced in upper extremities	No	No	No	No	No	No
Trigeminal SSEP	–	Normal	–	–	–	Normal
CSF abnormalities	Normal	Normal	Elevated protein	Elevated protein	Elevated protein	Normal
Deceased (age)	No	No	No	No	No	Yes (84)

*FEES* fiberoptic endoscopic evaluation of swallowing, *PEG* percutaneous endoscopic gastrostomy, *EMG* electromyography, *NCS* nerve conduction study, *SNAP* sensory nerve action potential, *CSF* cerebrospinal fluid

### Volumetry

At the group level, FOSMN patients showed a significant global volume reduction (atrophy) with the highest negative z scores in the medulla oblongata followed by the insula. In general, GM showed a higher volume reduction overall than WM in the lobes and in gyral GM, when compared to controls (Table 2).

**Table 2:**
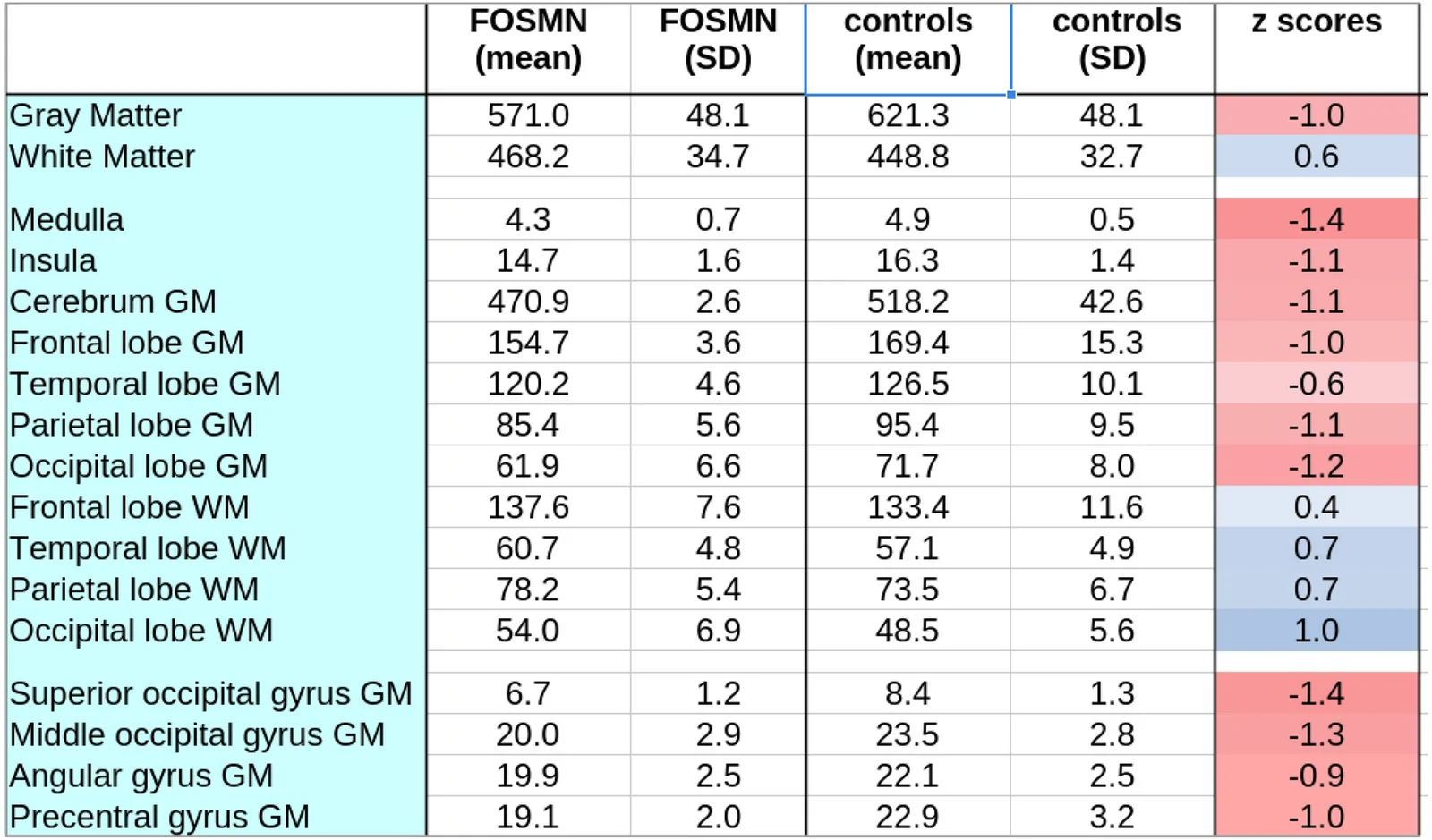
Atlas-based volumetry (ABV) results for white matter (WM) and grey matter (GM) structures based on T1-weighted imaging (group level analysis: FOSMN (n=6) vs 30 age-matched controls)

The z scores are color-coded, from blue (positive) to red (negative values)

### DTI

**Fig. 1:**
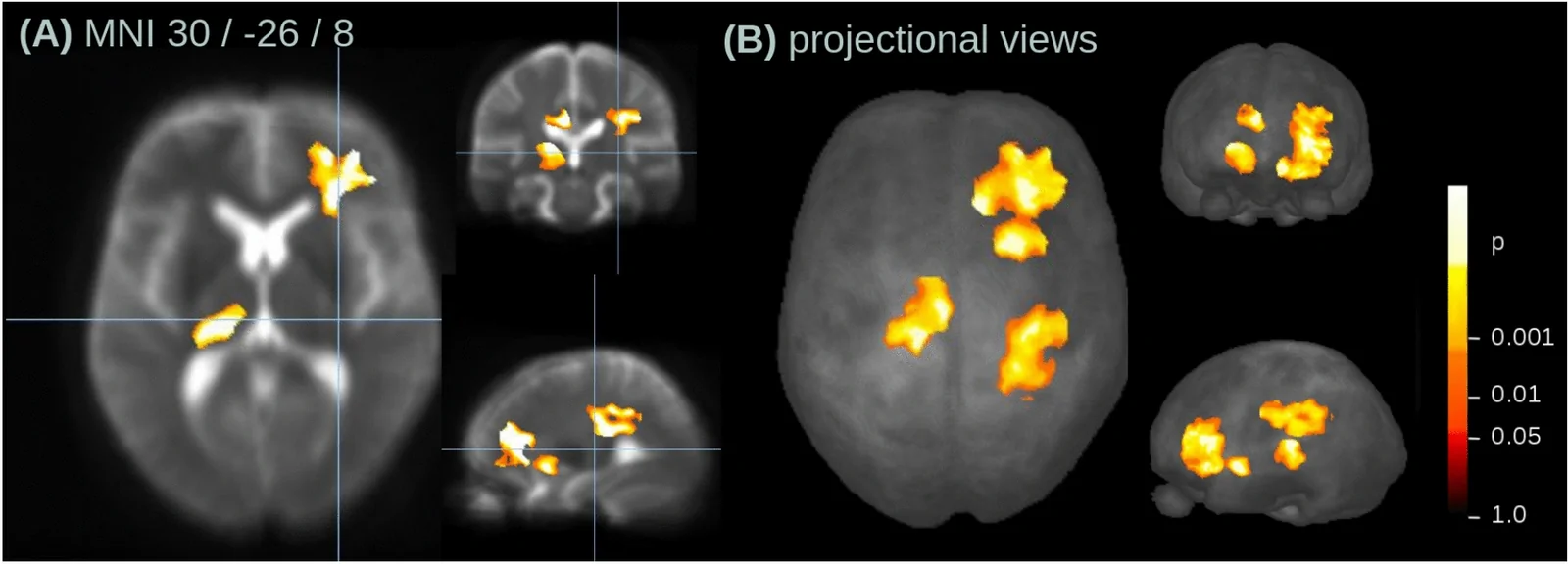
Whole brain-based spatial statistics (WBSS) for the comparison of FA maps of 6 patients with FOSMN vs 30 controls. Significance level is coded according to the color bar. Corrections for multiple comparisons were performed with the false discovery rate at p < 0.05 and a cluster-size approach. Details of the corresponding clusters are shown in Table 2. **A** Slicewise representation at Montreal Neurological Institute (MNI) coordinates (crosshair, x=30/y=− 26/z=8). **B** Projectional views

By use of WBSS for microstructural differences, the group differences in white matter FA values between 6 patients with FOSMN and 30 controls demonstrated a significant decrease in the right frontal lobe and bilaterally along both the superior and the inferior longitudinal fascicles (Fig. 1); the significant clusters are summarized in Table 3.

**Table 3:** Whole brain-based spatial statistics (WBSS) for cross-sectional comparison of FA maps of patients with FOSMN (n = 6) vs controls (n = 30); details of the clusters where FA changes were detected

Cluster size	Hemisphere	Anatomical location	MNI (x/y/z) of center of cluster	*p*
17,275	R	Frontal lobe	28/25/3	< 0.000001
‍8668	R	SLF	30/− 38/23	< 0.000001
3641	L	ILF	−17/− 34/3	< 0.000001
3200	L	SLF	−8/− 17/33	< 0.000001

*MNI* montreal neurological institute coordinate frame, *ILF* inferior longitudinal fasciculus, *SLF* superior longitudinal fasciculus

## Discussion

The etiology and the pathophysiology of FOSMN remain elusive, including the question whether the underlying mechanism may be neurodegenerative, given that it is an extremely rare neurological disease with a multifaceted clinical phenotype [11]. To this end, in vivo characterization of the pathoanatomical patterns by advanced neuroimaging may help guide the definitions of the disease process. In the current study, we used two unbiased MRI analysis approaches, i.e., ABV for volumetric patterns and DTI/WBSS for WM tract alterations, to identify structural and microstructural changes in the MRI datasets of a group of six FOSMN patients, representing the spectrum of the clinical phenotype. Despite the small sample size, ABV demonstrated a significant global atrophy with the most prominent findings in the medulla oblongata and, with respect to the cerebral GM, in the frontal and temporal lobes. DTI/WBSS identified a pattern with several significant clusters of substantial size along the SLF and ILF (bilaterally) and in the WM of the right frontal lobe.

This pattern of volumetric changes supports the view that FOSMN is a disorder involving large parts of the brain, as evidenced by global cerebral GM volume reduction. More specifically, brainstem areas (i.e., the medulla oblongata) are involved, where major nucleus regions of the trigeminal nerve, together with the medial lemniscus, are located. This volumetric pattern—combining global cerebral changes with a focus on the brainstem—is plausible for a disorder that affects various functional systems of the brain associated with cranial nerve V and bulbar involvement. With respect to WM tract structures, both the superior and the inferior longitudinal fascicles (SLF/ILF) demonstrated FA reductions, whereas there was no significant involvement of the corticospinal tract (CST). In addition, there was a large cluster of microstructural alterations in the frontal WM, similar to DTI-based findings in frontotemporal lobar degeneration (FTLD), such as alterations along the SLF and ILF [29]. This aligns with the general overlap between FOSMN and FTLD syndromes, which is also evident genetically [4, 7]. The SLF forms a large arcuate bundle that interconnects the frontal lobes with the post-Rolandic lobes and fans out into different parts of the temporal lobes, while the ILF constitutes an occipitotemporal projection system [30]. Direct connections to the face areas of the somatotopically structured human somatosensory cortex were not included in the significant clusters [31]; however, it is worth noting that affections of the trigeminal nerve (as in trigeminal neuralgia) have previously been associated with microstructural alterations of the SLF [32]. These findings of regional microstructural alterations support the view of FOSMN as a disorder which affects polymodal integration areas, providing a neuroanatomical correlate for the multifaceted clinical presentation. Concurrently, our data do not demonstrate a regional tract damage to the CST, as typically observed in motor neuron disease (MND) such as ALS. However, given the limited MND symptoms in this cohort, whether CST involvement occurs remains to be investigated in larger samples with more clinically pronounced motor neuron affection.

An obvious limitation of this study is the small sample size. However, against the background of about 100 cases reported worldwide so far, this number may be considered to be sufficient for a pilot study using a standardized advanced neuroimaging protocol on the same scanner. This constellation of significant volumetric findings alongside large clusters of regional WM alterations, even within this small sample, can be regarded as a strength, providing initial evidence for the potential of advanced MRI to define an in vivo neuroanatomical pattern for this rare disorder. Advanced MRI analysis techniques will be needed to address these aims, given that previous clinical reports had emphasized that the normal routine MRI studies in patients with FOSMN do not explain their symptoms [2]. Since DTI data cannot establish the directionality of tract alterations, these findings do not clarify the clinical rostro-caudal trajectory of symptoms. In addition, brain MRI cannot resolve the ongoing question of whether the nature of FOSMN is neurodegenerative or immune-mediated.

In summary, this first application of volumetry and DTI to a group of FOSMN patients demonstrated global brain atrophy with a regional focus in the medulla oblongata, together with tract alterations of polymodal frontotemporoparietal interconnections. This neuroanatomical pattern aligns with the clinical phenotype, characterized first by cranial nerve involvement at the brainstem level and second by a progression pattern involving different modalities including motor, somatosensory, and fronto-behavioral functions. Notably, the current data do not demonstrate a DTI-based pattern typical of motor neuron diseases like ALS, which characteristically involves the CST [33]—this absence of primary motor tract pathology is in line with the neurohistopathological findings that did not support a link to ALS in contrast to the existing clinical overlap [1, 2, 19]. However, CST involvement has to be further investigated in further MRI/DTI studies in patients with more prominent motor syndrome than our patients, since only one out of six patients in the current study showed upper motor neuron affection.

These signatures of GM atrophy and WM involvement contribute to the conceptual understanding of the processes that underlie this rare disorder; however, studies in larger cohorts, most likely in a multicenter design with standardized clinical and neuroimaging assessments, are needed to obtain sufficient sample sizes, which then could also incorporate volumetric and DTI-based data for the spinal cord. In particular, longitudinal studies evaluating phenotype–neuroimaging correlations of intraindividual symptom progression will potentially guide the pathophysiological understanding of FOSMN in the future.

## Data Availability

Reasonable data sharing requests can be addressed to the corresponding author and require a formal data sharing agreement with the University Hospital Ulm, Germany.
